# Supplementary material for: Phytochemical and Biological Evaluation of a Newly Designed Nutraceutical Self-Nanoemulsifying Self-Nanosuspension for Protection and Treatment of Cisplatin Induced Testicular Toxicity in Male Rats
Source: Molecules. 2021 Jan 14;26(2):408. doi: 10.3390/molecules26020408 (PMC7830605; doi:10.3390/molecules26020408)
Supplement: Supplementary file 1 [file molecules-26-00408-s001.pdf]

Figures:

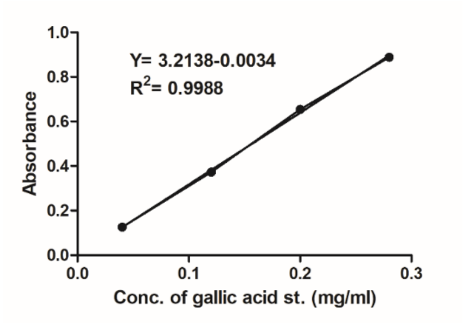

Figure S1. Standard calibration curve of gallic acid

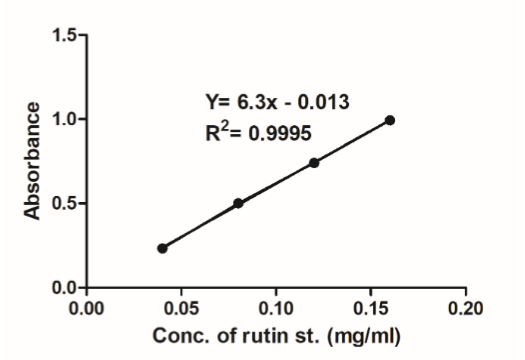

Figure S2. Standard calibration curve of rutin standard

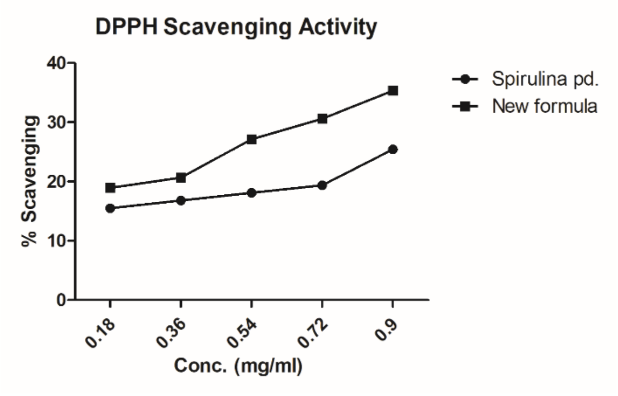

Figure S3. Scavenging ability of SP and NCF on DPPH radical

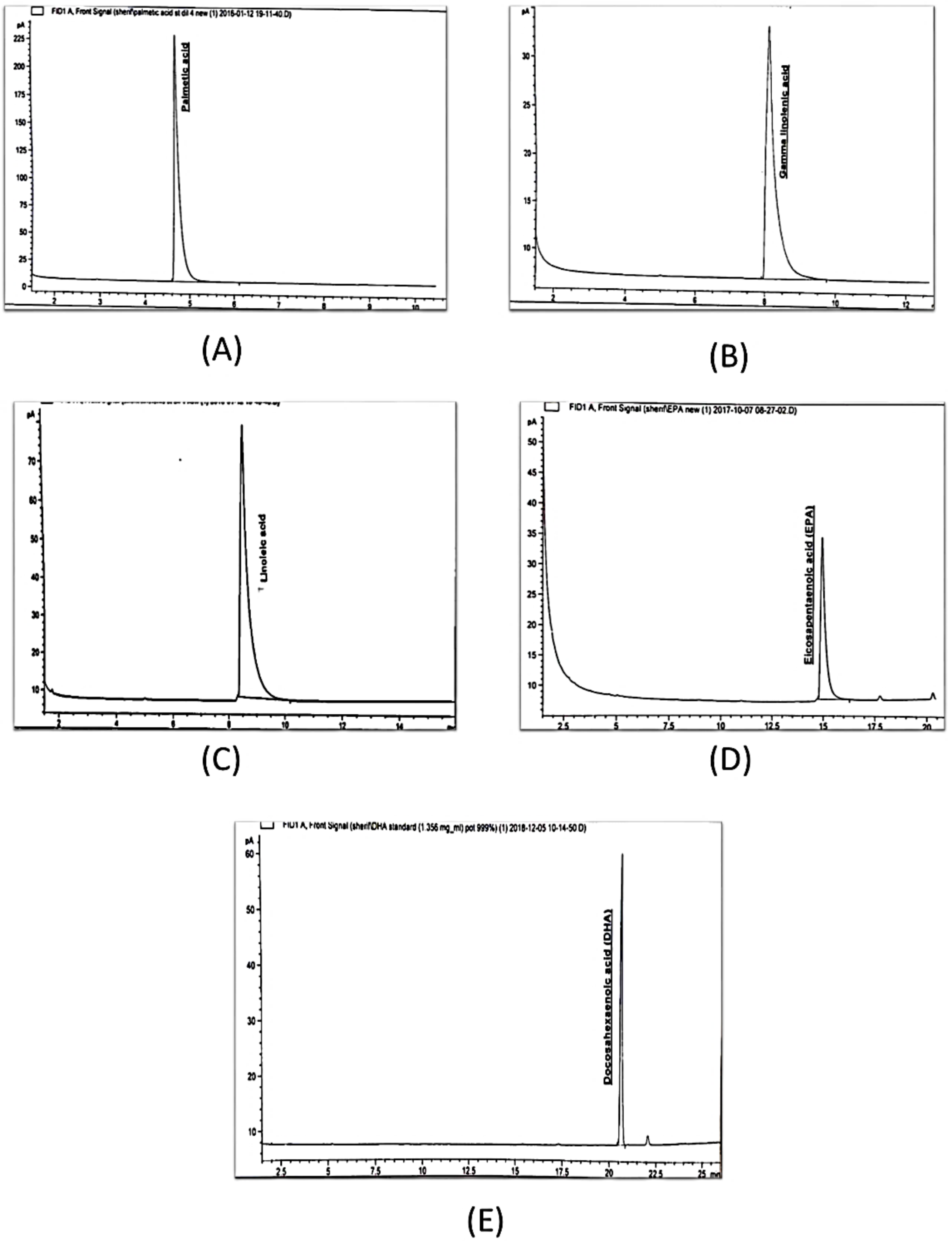

Figure S4. GC/FID chromatogram of fatty acid methyl esters of standards; (A): palmitic acid; (B): γ-linolenic acid; (C): linoleic acid; (D): eicosapentaenoic acid (EPA); (E): docosahexaenoic acid (DHA)

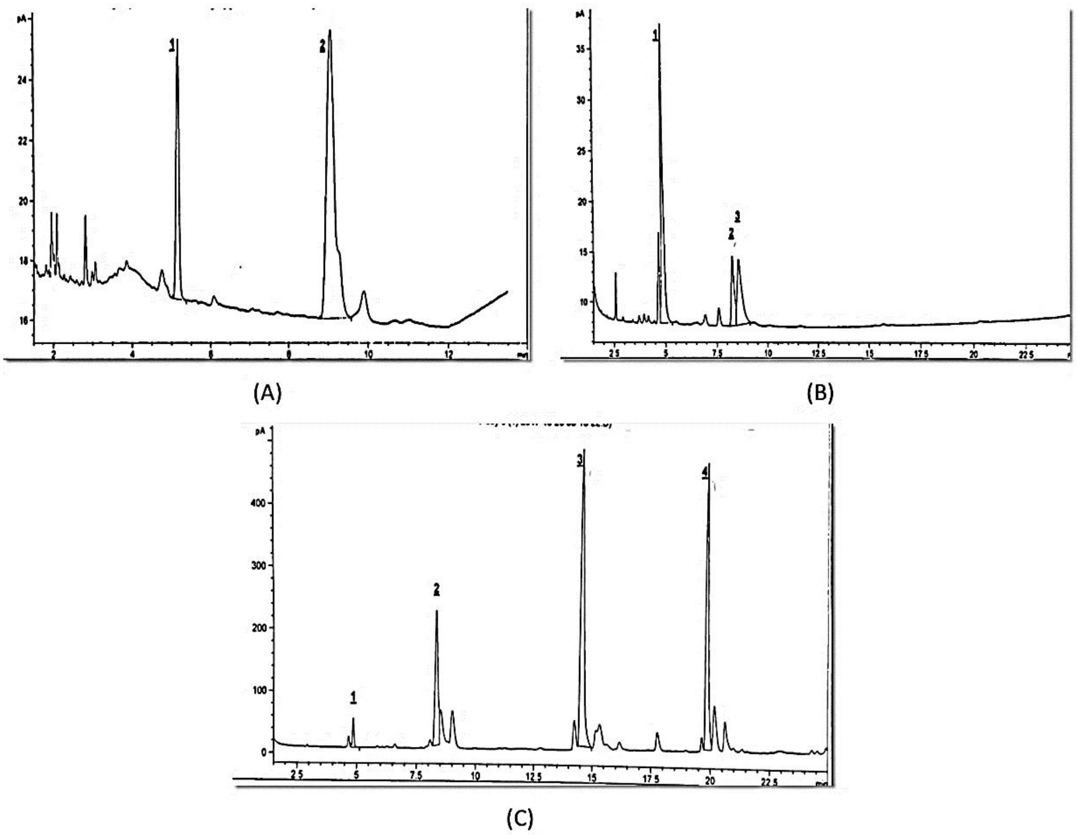

Figure S5. GC/FID chromatogram of fatty acid methyl esters of (A): TT; (B): SP; (C): NCF

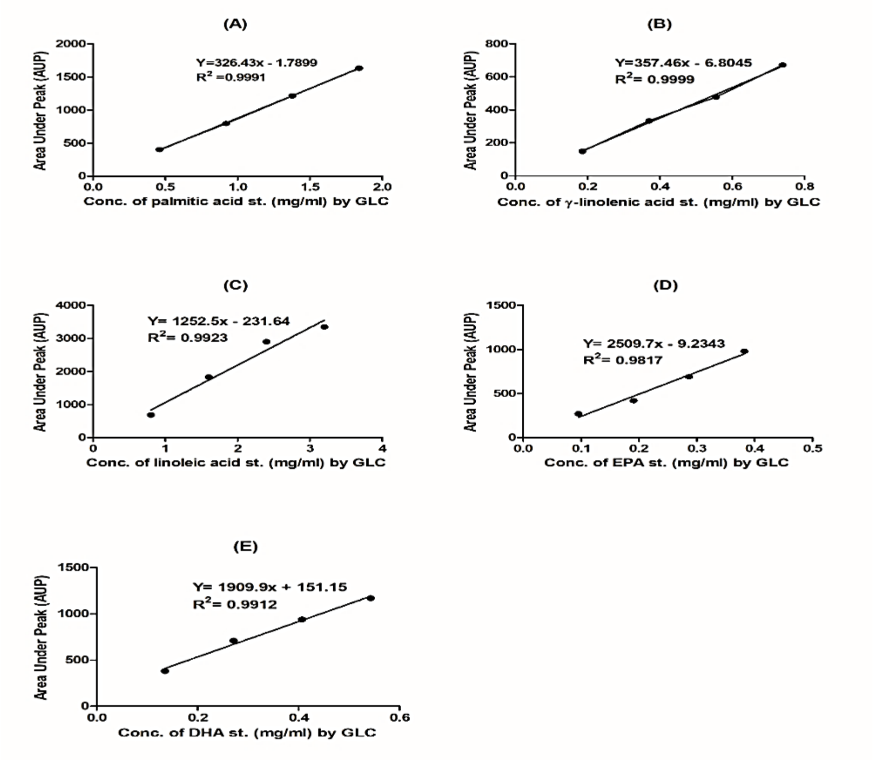

Figure S6. Standard calibration curve of reference standards of (A); palmitic acid, (B): γ- linolenic acid, (C): Linoleic acid; (D): eicosapentaenoic acid and (E): docosahexaenoic acid

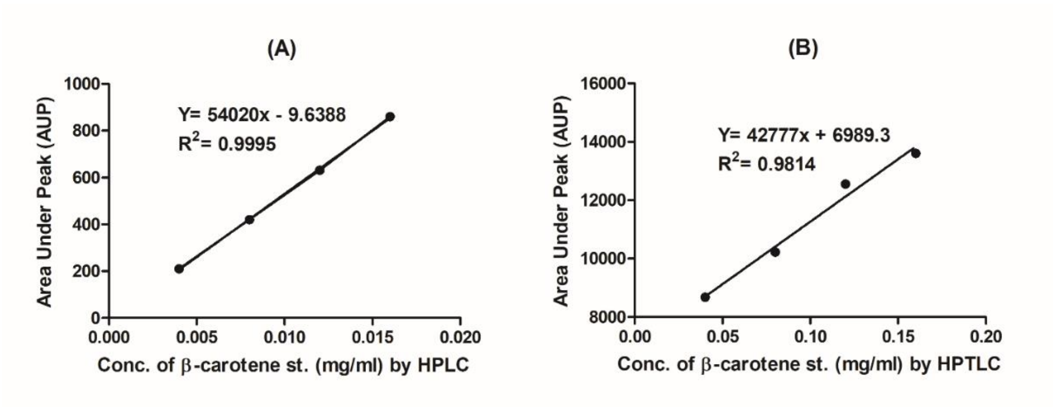

Figure S7. Standard calibration curve of *β*-carotene standard by; (A): HPLC and (B): HPTLC

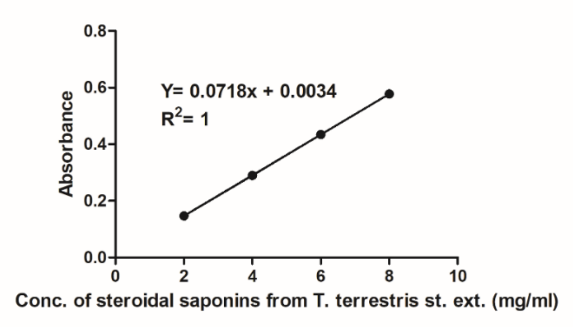

Figure S8. Standard calibration curve of steroidal saponins in TT working standard extract

<u>**Tables**</u>

Table S1. Antioxidant activity assayed by DPPH method of the SP and NCF (expressed as % Inhibition ± SD)

	**% Inhibition* ± SD**				
	**0.18 mg/ml**	**0.36 mg/ml**	**0.54 mg/ml**	**0.72 mg/ml**	**0.9 mg/ml**
**SP**	15.517 ± 0.00208	16.81 ± 0.00151	18.103 ± 0.00321	19.396 ± 0.002	25.431 ± 0.00321
**NCF**	18.965 ±0.0035	20.689 ±0.0084	27.155 ±0.0021	30.603 ±0.0065	35.344 ±0.0073

*Average of the three determinations.

Table S2. Antioxidant activity assayed by DPPH test of the SP and NCF (expressed as IC_{50} ± SD)

	**IC_{50} (mg/ml)* ± SD**
**SP**	1.606 ± 0.026
**NCF**	1.122 ± 0.038
**Gallic acid**	0.016 ± 0.002

*Average of the three determinations.

Table S3. Results for the analysis of vitamin B_{12}, C and E in the SP

**Vitamin**	**Concentration (mg/100 gm)**	**RDA (Recommended Dietary Allowances) (www.ods.od.nih.gov)**
**Vitamin B_{12}**	3.2	2.4 µg
**Vitamin C**	191.906	90 mg

**Vitamin E**	undetectable	g

Table S4. Results of analysis of minerals content of SP

Minerals	Conc (mg/100gm)	RDA (Recommended Daily Allowance) [86]
**Iron (Fe)**	170	8 mg
**Zinc (Zn)**	160	11 mg
**Copper (Cu)**	20	900 µg

Table S5. Composition of ternary mixtures

Formula code	% Fish oil	% Surfactant (Span 80/Cremophore EL)	% Co-surfactant (Isopropyl alcohol)
F1	50	40	10
F2	50	30	20
F3	50	20	30
F4	40	40	20
F5	40	30	30
F6	40	20	40
F7	35	60	5
F8	35	50	15
F9	35	40	25
F10	30	60	10
F11	30	50	20
F12	30	40	30

Table S6. A. GC/FID investigation of FAME standards, SP, TT, FO and NCF

Sample	Peak area	Conc.
**Palmitic acid standard solution (A)**	402.405	0.46 mg/ml
**Palmitic acid standard solution (B)**	796.054	0.92 mg/ml
**Palmitic acid standard solution (C)**	1216.136	1.38 mg/ml
**Palmitic acid standard solution (D)**	1634.295	1.84 mg/ml
**γ-linolenic acid standard solution (A)**	148.56	0.185 mg/ml
**γ-linolenic acid standard solution (B)**	333.66	0.37 mg/ml
**γ-linolenic acid standard solution (C)**	477.531	0.555 mg/ml
**γ-linolenic acid standard solution (D)**	671.928	0.74 mg/ml
**Linoleic acid standard solution (A)**	685.3895	0.8 mg/ml
**Linoleic acid standard solution (B)**	1836.0861	1.6 mg/ml
**Linoleic acid standard solution (C)**	2901.8483	2.4 mg/ml
**Linoleic acid standard solution (D)**	3349.0089	3.2 mg/ml
**EPA standard solution (A)**	270.506205	0.0956 mg/ml
**EPA standard solution (B)**	420.89235	0.1912 mg/ml
**EPA standard solution (C)**	690.5709	0.2868mg/ml
**EPA standard solution (D)**	980.37345	0.3824 mg/ml
**DHA standard solution (A)**	380.90286	0.1356 mg/ml
**DHA standard solution (B)**	710.5096	0.2712 mg/ml
**DHA standard solution (C)**	940.5693	0.4068 mg/ml
**DHA standard solution (D)**	1170.0191	0.5424 mg/ml

Table S6. B.

Sample	Palmitic acid area	GLA area	Linoleic acid area	EPA area	DHA area
**SP**	860.103	413.152	464.457	-	-
**TT**	12.058	-	27.123	-	-
**FO**	179.368	-	1609.34	4768.28	3206.27
**NCF**	2979.666	523.84	10529.3	4851.87	3273.92

Table S7. Fatty acid methyl esters concentration in SP, TT, FO and NCF

	Spirulina Powder	*T. terrestris* ext.	Omega-3 fish oil	New designed formula
**% Palmitic acid**	13.2% ± 0.14	2.12% ± 0.25	6.937% ± 0.77	27.67% ± 0.45
**Conc. (mg/gm)**	132 mg/gm pd.	21.2 mg/gm ext.	69.37 mg/gm oil	110.68 mg/gm form.
**% GLA**	5.874% ± 0.02	-	-	6.747% ± 1.72
**Conc. (mg/gm)**	58.74 mg/gm pd.	-	-	16.8675 mg/gm form.
**% Linoleic acid**	2.77% ± 0.08	10.32% ± 0.7	18.373% ± 0.33	26.03% ± 0.99
**Conc. (mg/gm)**	27.7 mg/gm pd.	103.2 mg/gm ext.	183.73 mg/gm oil	104.12 mg/gm form.
**% EPA**	-	-	23.795% ± 1.42	22.04% ± 0.56
**Conc. (mg/gm)**	-	-	237.92 mg/gm oil	22.04 mg/gm form.
**% DHA**	-	-	19.99% ± 0.99	18.58% ± 0.08
**Conc. (mg/gm)**	-	-	199.9 mg/gm oil	18.58 mg/gm form.

Table S8. Method validation and statistical analysis of FAME in SP, FO, TT and NCF by GC/FID

	**Item**		**Palmitic acid**	**GLA**	**Linoleic acid**	**EPA**	**DHA**
**Linearity**	**Regression equation**		Y = 326.43x – 1.7899	Y = 357.46x – 6.8045	Y = 1252.5x – 231.64	Y = 2509.7x – 9.2343	Y = 1909.9x + 151.15
	**Calibration range (mg/ml)**		0.46 – 1.84	0.185 – 0.74	0.8 – 3.2	0.1114 – 0.779	0.0966 – 0.676
	**Correlation coefficient (R^2)**		0.9991	0.9999	0.923	0.9817	0.9912
	**%RSD**		1.367	1.233	1.248	1.512	1.88
**Accuracy**	**Recovery percent**		99.88%`	99.88%	97.05%	101.45%	99.415%
**Precision**	**Intra-day precision (%RSD, n = 3)**	**SP**	0.15%	0.02%	0.0636%	-	-
		**FO**	2.62%	-	0.01%	1.11%	0.46%
		**TT**	0.26%	-	0.06%	-	-
		**NCF**	0.45%	1.24%	0.964%	1.93%	2.5%
	**Inter-day precision (%RSD, n = 3)**	**SP**	4.04%	6.23%	3.28%	-	-
		**FO**	6.6%	-	12.8%	1.48%	1.9895%
		**TT**	13.34%	-	4.74%	-	-
		**NCF**	0.497%	2.09%	1.91%	1.75%	1.78%
**Sensitivity**	**Detection limit (LOD) (mg/ml)**		0.0986	0.137	0.568	0.0053	0.0132
	**Quantification limit (LOQ) (mg/ml)**		0.298	0.416	1.721	0.016	0.04

Table S9. HPLC investigation of *β*-carotene in SP and NCF

Sample	AUP	Concentration
**β-Carotene standard solution (A)**	210.41	0.004 mg/ml
**β-Carotene standard solution (B)**	420.4145	0.008 mg/ml
**β-Carotene standard solution (C)**	630.903	0.012 mg/ml
**β-Carotene standard solution (D)**	860.512	0.016 mg/ml
**SP**	468.242	0.008846 mg/ml
**NCF**	177.236	0.003459 mg/ml

Table S10. Concentration of β-carotene in SP and NCF by HPLC

Sample	RT	Peak Area	Concentration
**SP**	7.28	468.242	0.44 mg β-carotene/ g powder
**NCF**	7.31	177.236	0.17 mg β-carotene/ g formula

Table S11. HPTLC investigation of β-carotene in SP and NCF

Sample	AUP	Concentration
**β-carotene standard solution (A)**	8679.5	0.04 mg/ml
**β-carotene standard solution (B)**	10224.67	0.08 mg/ml
**β-carotene standard solution (C)**	12559	0.12 mg/ml
**β-carotene standard solution (D)**	13605	0.16 mg/ml
**SP**	8378	0.0324 mg/ml

Table S12. Concentration of β-carotene in SP and NCF by HPTLC

Sample	RF	Peak Area	Concentration
**SP**	0.87	8378	1.63 mg β-carotene/ g powder

Table S13. Method validation and statistical analysis of β-carotene in SP and NCF by HPLC, HPTLC

Item			HPLC	HPTLC
**% β-carotene in SP**			0.044%	0.162%
**% β-carotene in NCF**			0.058%	-
**Linearity**	**Regression equation**		Y = 54020x – 9.6388	y = 42777x + 6989.3
	**Calibration range (mg/ml)**		0.004 – 0.016	0.04 – 0.16
	**Correlation coefficient, R^2**		0.9995	0.9814
**Accuracy**	**Recovery percent**		100.2051%	99.62%
**Relative standard deviation (%RSD, n=3)**			0.32%	1.679
**Sensitivity**	**Detection limit (LOD) (mg/ml)**		0.46 μg/ml	0.0287 mg/ml
	**Quantification limit (LOQ) (mg/ml)**		1.3942 μg/ml	0.0871 mg/ml
**Precision**	**Intra-day precision (%RSD, n=3)**	**SP**	0.56%	2.79%
		**NCF**	0.03%	-
	**Inter-day precision (%RSD, n=3)**	**SP**	54.67%	60%
		**NCF**	6.2%	-

Table S14. Method validation and statistical analysis of β-carotene in SP and NCF by HPLC, HPTLC

Sample	Absorbance	Concentration
**Steroidal saponins solution (A)**	0.147333	2 mg/ml

**Steroidal saponins solution (B)**	0.289667	4 mg/ml
**Steroidal saponins solution (C)**	0.434333	6 mg/ml
**Steroidal saponins solution (D)**	0.5775	8 mg/ml
**TT**	0.295	4.0612 mg/ml
**NCF**	0.129	1.75 mg/ml

Table S15. Method validation and statistical analysis of total steroidal saponins in TT and NCF determined by the U.V colorimetric method

**Item**			**Result**
**Linearity**	**Regression equation**		Y = 0.0718x + 0.0034
	**Calibration range (mg/ml)**		2-8
	**Correlation coefficient, R^2**		1
**Accuracy**	**Recovery percent**		99.97%
**Relative standard deviation (%RSD, n=3)**			1.6%
**Sensitivity**	**Detection limit (LOD) (mg/ml)**		0.03097%
	**Quantification limit (mg/ml)**		0.09387%
**Precision**	**Intra-day precision (%RSD, n=3)**	**TT**	0.91%
		**NCF**	0.48%
	**Inter-day precision (%RSD, n=3)**	**TT**	17.22%
		**NCF**	18.18%

Table S16. Effect of 20 days' administration of TT (100 mg/kg/orally), SP (1000 mg/kg/orally), FO (400 mg/kg/orally) and (4.4 g/kg/orally) NCF on CP-induced alterations in spermatological parameters

**Groups**	**NC**	**CP**	**TT**	**SP**	**FO**	**NCF**
**Parameters**						
**Sperm count (10^6/ml)**	266 ± 6.38	60 ± 5.28	192 ± 7.54	236 ± 5.28@	93 ± 5.28*#	260 ± 4.41@
**Sperm abnormalities (%)**	16 ± 3.306	31.67 ± 5	17.33 ± 0.5774	17 ± 2.616 @	23 ± 2 *@#	16.67 ± 1.258@
**Individual motility (%)**	69.3 ± 7.638	35 ± 5	60 ± 5	65 ± 2.887@	40 ± 5 *#	75 ± 2.887@
**Life-dead (%)**	86.67 ± 4.163	41.07 ± 1.41	71.67 ± 2.887	75 ± 5@	53.33 ± 7.638 *@#	84.53 ± 4.041@

Values are means of 6-10 rats ± SD. Statistical analysis was performed using one-way ANOVA followed by Newman Keuls multiple comparison test (*P*< 0.05). As compared with normal control (NC; *), cisplatin (CP; @), Tribulus terrestris (TT; #), Spirulina platensis (SP), Fish oil (FO).

Table S17. Biochemical investigation of the effect of TT, SP, FO and HF against serum testosterone, testicular oxidative stress and antioxidant markers (tissue MDA, NO, GSH, TAC and Nrf2), testicular inflammatory status (IL-6 and NFkB) and apoptotic status (caspase-3)

**Parameter**	**CP Treatment**					
	**NC**	**CP**	**TT**	**SP**	**FO**	**NCF**
**Serum Testosterone (ng/ml)**	4.807 ± 0.3274	1.023 ± 0.241*	1.873 ± 0.1650 *	2.270 ± 0.3179*, @	1.927 ±0.2293 *	3.520 ± 0.1721*,@,#
**Tissue IL-6 (ng/mg)**	3.740 ± 0.3400	13.66 ± 1.474*	10.76 ± 1.661*	10.03 ± 0.3283*	11.93 ± 1.169*	6.750 ± 0.8109@
**Tissue Caspase-3 (ng/mg)**	2.834 ± 0.2407	6.114 ± 0.3843 *	4.182 ± 0.1885 *, @	3.2 ± 0.2082 @	3.948 ± 0.1523 *, @	2.643 ± 0.1767 @, #

**Tissue NFκB (ng/mg)**	1.046 ± 0.1272	6.270 ± 0.2973 *	3.960 ± 0.2977 *, @	2.830 ± 0.2150 *, @, #	2.150 ± 0.1708 *, @, #	1.713 ± 0.1793 @, #
**Tissue Nrf2 (ng/mg)**	442.0 ± 35.16	108.4 ± 7.774 *	203.0 ± 26.24 *, @	314.3 ± 25.76 *, @, #	368.8 ± 20.80 *, @, #	481.3 ± 21.47 @, #
**Tissue MDA**	0.3236 ± 0.01722	1.999 ± 0.1675 *	1.064 ± 0.1934 *, @	0.6360 ± 0.1094 @	0.6628 ± 0.07066 @	0.3798 ± 0.04617 @, #
**Tissue GSH**	554.0 ± 23.69	111.8 ± 22.39 *	161.2 ± 17.26 *	374.0 ± 54.93 *, @, #	259.0 ± 17.21 *, @, #	331.5 ± 15.82 *, @, #
**Tissue TAC**	317.0 ± 33.38	119.5 ± 23.68 *	171.2 ± 20.44 *	275.7 ± 10.35 @, #	186.8 ± 29.95 *	319.0 ± 23.40 @, #
**Tissue NO**	608.8 ± 93.05	184.8 ± 18.78 *	200.2 ± 35.84 *	284.0 ± 42.44 *	232.8 ± 26.66 *	378.3 ± 25.71 *, @, #

All numbers are mean + standard error, n = 8

*Significantly different from control value, *P < 0.05.

@Significantly different from cisplatin group value, @P < 0.05.

#Significantly different from reference drug (TT) group value, #P < 0.05.

NC: normal control; CP: positive control (cisplatin); TT: Reference drug (Tribulus terrestris extract); SP: spirulina powder; FO: omega-3 fish oil and NCH: Novel Reference Drug
